# Peg-IFNα reduces clinical severity but cannot prevent COVID-19 infection in chronic hepatitis B patients: a cohort study in China

**DOI:** 10.3389/fmed.2026.1843714

**Published:** 2026-07-08

**Authors:** Tiantian Hu, Weien Yu, Jie Tong, Yunhui Yang, Changrong Yuan, Richeng Mao, Jiming Zhang, Jinyu Wang

**Affiliations:** 1 Shanghai Key Laboratory of Infectious Diseases and Biosafety Emergency Response, Department of Infectious Diseases, National Medical Center for Infectious Diseases, Huashan Hospital, Fudan University, Shanghai, China; 2 Key Laboratory of Medical Molecular Virology (MOE/MOH), Shanghai Institute of Infectious Diseases and Biosecurity, Shanghai Medical College, Fudan University, Shanghai, China; 3 Fudan University School of Nursing, Fudan University, Shanghai, China; 4 Department of Infectious Diseases, Jing’An Branch of Huashan Hospital, Fudan University, Shanghai, China

**Keywords:** CHB, clinical severity, HBV, PEG-IFN-α-2a, SARS-CoV-2

## Abstract

**Introduction:**

SARS-CoV-2/HBV coinfections are increasingly common as SARS-CoV-2 continues to evolve. The interferon (IFN) therapy for HBV infection might also have antiviral effects against SARS-CoV-2. To understand the effects of IFN on SARS-CoV-2 infection, we compared the clinical characteristics between IFN-treated chronic hepatitis B (CHB) patients and nucleo(s)tide analogs (NAs)-treated CHB patients during their SARS-CoV-2 infection.

**Methods:**

Our cohort study enrolled patients with primary SARS-CoV-2 infections among IFN-treated and NAs-treated CHB patients during the Omicron wave in Shanghai, China, from December 7 2022 to January 23 2023. Clinical characteristics and self-reported acute symptoms during SARS-CoV-2 infection were collected by telephone questionnaire in January 2023.

**Results:**

The four most common symptoms were fever, fatigue, cough and sore throat among all enrolled participants, regardless of their treatment. Interestingly, Pegylated interferon-alpha (Peg-IFNα) treated CHB patients had a significantly higher proportion of asymptomatic infection than NAs-treated CHB patients (14.53% vs. 2.70%, *p* = 0.002). Additionally, IFN-treated CHB patients had significantly lower duration of fever (*p* = 0.018), less usage of antipyretics (*p* < 0.0001), less occurrence of muscle and/or joint pain (*p* < 0.0001), less occurrence of headache (*p* < 0.0001) and less occurrence of runny nose (*p* < 0.0001) compared to NAs-treated CHB patients.

**Conclusion:**

In this real-world cohort of CHB patients infected with SARS-CoV-2 during the Omicron wave, Peg-IFNα treatment was associated with milder self-reported acute clinical manifestations, including a higher proportion of asymptomatic infection and reduced occurrence of several common symptoms.

## Introduction

COVID-19, an infectious disease caused by severe acute respiratory syndrome coronavirus 2 (SARS-CoV-2), has resulted in over 770 million confirmed cases and over 6.9 million deaths as of 27 August 2023 (1). SARS-CoV-2 infection rates are continuously rising as the COVID-19 pandemic persists and the number of people with prior infection grows (2). Shanghai suffered the second Omicron wave dominated by BA.5.2 and BF.7 since 7 December 2022, when China started to apply lax anti-COVID-19 measures (3–5). Notably, due to the high prevalence of HBV infection in China, SARS-CoV-2/HBV coinfection is becoming more common (6–8).

As an immune-based therapy, interferon-alpha (IFN-α) has been widely used for chronic hepatitis B (CHB) and can lead to HBsAg loss in 20–30% of treated patients (9). IFN therapy has also been used for COVID-19 patients, leading to shorter viral clearance, reduced systemic inflammation, and lower death rates (10, 11). Usage of IFN in combination with other traditional antiviral therapies to treat COVID-19 has also been reported (12). Multiple studies have suggested that a deficiency in IFN-α levels may be associated with the severity of COVID-19 and indicated that immune pathways could serve as potential targets for therapeutic intervention (13–15). However, the broad application of IFN-α in COVID-19 therapy remains controversial due to its side effects, including flu-like symptoms, hematological abnormalities, and neuropsychiatric issues, which could limit its long-term applicability (16). Balancing the antiviral property of IFN with its significant side effects would be critical to determining its further role in COVID-19 treatment (17–19). Moreover, IFN-α has proven effective in treating viral infections such as SARS-CoV and MERS-CoV by stimulating both innate and adaptive immune responses (20, 21). The potential protective role of IFN therapy in COVID-19 remains controversial and warrants further investigation.

To further explore the clinical characteristics of SARS-CoV-2/HBV coinfection and the effect of IFN therapy in COVID-19, we conducted a cohort study. We enrolled CHB patients infected with SARS-CoV-2 during the second Omicron wave in Shanghai from December 7, 2022 to January 23, 2023 (22). Participants were divided into two groups based on their therapy for CHB: one group included CHB patients treated with IFN-α, and the other included CHB patients treated with nucleo(s)tide analog (NAs). All basic information was collected from Huashan Hospital.

## Materials and methods

### Study design

We conducted a real-world cohort study of CHB patients who experienced their first confirmed SARS-CoV-2 infection between December 7, 2022, and January 23, 2023, during the Omicron wave in Shanghai (22). Based on their antiviral regimen, participants were categorized into two groups: those receiving interferon-alpha (IFN-α) and those on nucleos(t)ide analogs (NAs), as previously described (23). IFN-treated CHB patients were followed up every 2 weeks at outpatient clinic of Huashan Hospital, while NAs-treated CHB patients were followed up monthly. Follow-up assessments were conducted throughout the study period.

Basic information on study participants was obtained from Huashan Hospital. Clinical characteristics and self-reported acute symptoms during SARS-CoV-2 infection, including symptom duration and medication use, were collected by telephone questionnaire in January 2023. SARS-CoV-2 infection was defined as SARS-CoV-2 antigen detection positive or nucleic acid test positive (19). This study was approved by the Ethics Committee of Huashan Hospital (protocol number: KY2022-721).

### Inclusion and exclusion criteria

For the group of IFN-treated CHB patients, the inclusion criteria included: 1) CHB patients who had HBV infection for over 6 months with positive HBV DNA or hepatitis B surface antigen (HBsAg); 2) Age ≥ 18 years old; 3) Treated with PEG-IFNα for over 6 months; 4) Infected with SARS-CoV-2 for the first time during December 7, 2022 to January 23, 2023; 5) Volunteer to join the study.

For the group of NAs-treated CHB patients, the inclusion criteria included: (1) CHB patients who had HBV infection for over 6 months with positive HBV DNA or HBsAg; (2) Age ≥ 18 years old; (3) Treated with *NAs* for over 6 months, including tenofovir alafenamide fumarate (TAF), tenofovir disoproxil fumarate (TDF), tenofovir amibufenamide (TMF) and entecavir (ETV) (24); (4) Infected with SARS-CoV-2 for the first time during December 7, 2022 to January 23, 2023; (5) Volunteer to join the study.

The exclusion criteria included: (1) Did not know if they were infected with SARS-CoV-2; (2) Unable to answer the questions due to serious illness; (3) Unwilling to join the study.

### Data collection

The basic information including gender, age, type of COVID-19 vaccine, dose of vaccination, date of SARS-CoV-2 infection, clinical characteristics of infection (including occurrence and duration of symptom, fever, fatigue, sore throat, cough, anosmia or ageusia, palpitation, muscle and/or joint pain, diarrhea or vomit, and so on) of all enrolled subjects were recorded. Cirrhosis status and major comorbidities, including hypertension, diabetes mellitus, cardiovascular disease, dyslipidemia, and fatty liver, were retrospectively extracted from electronic medical records and included as baseline clinical variables. HBV DNA levels at the time of SARS-CoV-2 infection, cumulative Peg-IFNα dose, time to SARS-CoV-2 RNA clearance, and inflammatory markers such as CRP and IL-6 were not systematically available for all participants and therefore were not included in the present analysis.

The questionnaire assessed 18 commonly reported symptoms during SARS-CoV-2 infection. It was designed to capture common self-reported acute clinical manifestations rather than the full spectrum of all possible COVID-19-related manifestations involving different body systems. The questionnaire used in this study was self-developed specifically for the purpose of assessing SARS-CoV-2 clinical manifestations among CHB patients. An English-language version has been provided as Supplementary File 1. The severity is calculated according to the mean ± SD or median (IQR) of the patients’ symptom self-assessment score. Each symptom was graded as absent, mild, moderate, or severe and assigned a score of 0–3. Vaccination status was categorized into three groups, “not fully vaccinated,” “fully vaccinated,” and “boostered.” The “not fully vaccinated” group included those with no COVID-19 vaccines or an incomplete primary vaccination per local guidelines. “Fully vaccinated” referred to individuals who completed the primary series at least 14 days before assessment, in line with local policies. The “boostered” group consisted of people who had at least one additional booster dose beyond the primary series, with the last dose taken ≥ 14 days prior to assessment. These vaccination status definitions followed the national public health criteria during the study. Asymptomatic infection (“asymptomatic”) was defined as confirmed COVID-19 patients who developed no symptoms during infection. “With pneumonia” was defined as symptomatic infection with CT-confirmed pneumonia and was considered compatible with moderate or more severe COVID-19. Symptomatic infection without CT-confirmed pneumonia was considered clinically consistent with mild COVID-19. Because respiratory rate, oxygen saturation, PaO_2_/FiO_2_, ICU admission, and mechanical ventilation were not systematically available for all participants, patients with pneumonia could not be further classified into moderate, severe, and critical categories. Occurrence of sore throat was categorized into four groups: “no” referred to having no sore throat, “mild” was defined as tolerate pain, “severe” was defined as cutting pains which were unbearable, and “moderate” was defined as between mild and severe. Occurrence of cough was categorized into four groups: “no” referred to having no cough, “mild” was defined as cough occasionally, “severe” was defined as a frequent cough and continues at night, and “moderate” was defined as between mild and severe. “Pneumonia” was defined as showing signs of pneumonia by CT scan of the lungs, which was recommended for patients having consistent cough over a week, having fever over 38.5°C for over 3 days, or with oxygen saturation index < 93%. The questionnaire focused on self-reported acute clinical manifestations during SARS-CoV-2 infection. Post-COVID condition or long COVID was not systematically assessed, because symptoms were not collected at prespecified time points after recovery.

### Statistical analyses

All analyses were undertaken using R version 4.1.1 (R Foundation for Statistical Computing, Vienna, Austria) and RStudio (Posit, Boston, United States). Continuous variables were expressed as mean ± standard deviation for normally distributed data or median (interquartile range) for non-normally distributed data. Data distribution normality was evaluated using the Kolmogorov-Smirnov and Shapiro-Wilk tests. For categorical data comparisons across groups, we applied the Chi-square test or Fisher’s exact probability test where appropriate. When data met the assumptions of normality and homogeneity of variance, the independent cohort *t*-test was utilized for comparisons. In cases of non-normal distribution, the Mann-Whitney U test was employed instead. A *p*-value of < 0.05 was considered to determine statistical significance.

## Results

### Characteristics of IFN-treated CHB patients and NAs-treated CHB patients infected with SARS-CoV-2 during December 2022-January 2023

**FIGURE 1:**
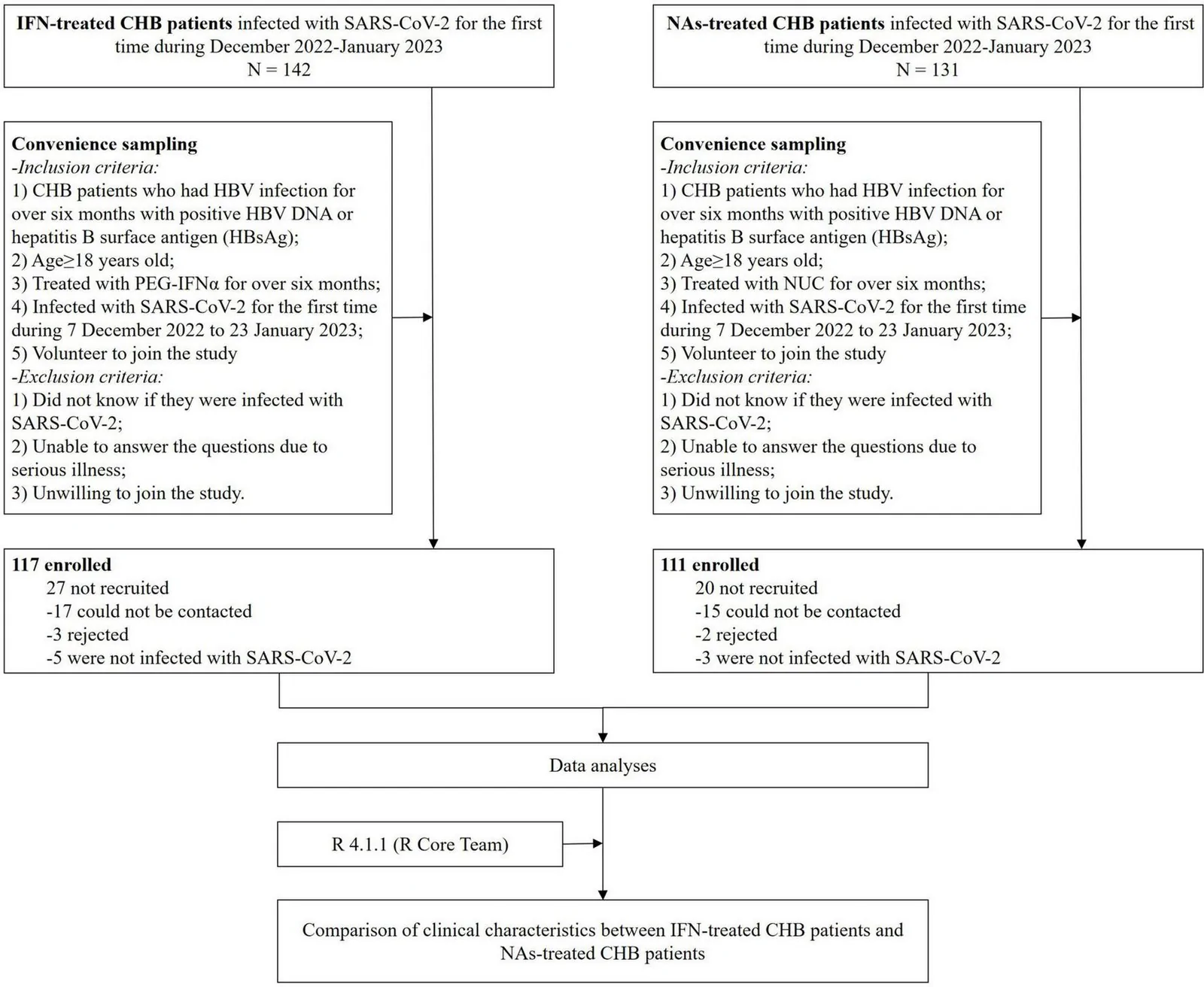
Flow diagram of subject enrollment and study design. The flow diagram illustrates the process of subject enrollment and study design for comparing clinical characteristics between IFN-treated CHB patients and NAs-treated CHB patients infected with SARS-CoV-2 for the first time during December 7, 2022, to January 23, 2023. The study included a total of 142 IFN-treated CHB patients and 131 NAs-treated CHB patients. Data analyses were performed using R 4.1.1. Statistical methods such as the Kolmogorov-Smirnov test and Shapiro-Wilk test were used to assess data normality. Chi-square test or Fisher’s exact probability test was applied to compare enumeration data between groups. For quantitative data, independent cohort *T*-test was used for normally distributed data with homogeneity of variance, while Mann-Whitney U test was used for skewed data. A *p*-value < 0.05 was considered statistically significant. CHB, Chronic Hepatitis B; IFN, interferon; PEG-IFNα, Pegylated Interferon alpha; NAs, nucleoside analogs.

Among 142 contacted IFN-treated CHB patients, 125 responded (response rate is 88.03%), 5 of them were not infected with SARS-COV-2, and 3 of them rejected the enrollment. Finally, 117 IFN-treated CHB patients infected with SARS-CoV-2 were enrolled (Figure 1 and Table 1). Among 131 contacted NAs-treated CHB patients, 116 responded (response rate is 88.55%), 3 of them were not sure whether they were infected with SARS-CoV-2 or not, and 2 of them were rejected the enrollment. A total of 111 NAs-treated CHB patients infected with SARS-CoV-2 were enrolled (Figure 1). There was no statistical difference in gender, vaccination status, type of vaccine, heterologous vaccination, or date of last vaccination between the two groups of CHB patients. Additional baseline variables were further compared. HBsAg levels were not significantly different between the Peg-IFNα and NAs groups [167.15 (26.23, 693.57) vs. 321.34 (46.06, 998.03) IU/mL, *p* = 0.052]. Similarly, no significant differences were observed in cirrhosis, hypertension, diabetes mellitus, cardiovascular disease, or fatty liver. Dyslipidemia was more frequent in the Peg-IFNα group than in the NAs group [19/117 (16.24%) vs. 7/111 (6.31%), *p* = 0.022] (Table 1).

**TABLE 1:** Clinical characteristics of all enrolled CHB patients infected with SARS-CoV-2 for the first time during December 2022 to January 2023.

Category	IFN-treated CHB patients (*n* = 117)	NAs-treated CHB patients (*n* = 111)	*P*-value
Gender			0.089
Male	94 (80.35)	78 (70.27)	0.091
Age group (years)			**0.007**
<18	0	0	1
18–40	67 (57.26)	80 (72.07)	**0.027**
41–60	49 (41.88)	27 (24.32)	**0.005**
61–75	1 (0.86)	4 (3.61)	0.203
> 75	0	0	1
Vaccination status			0.894
Not fully vaccinated	13 (11.11)	11 (9.91)	0.683
Fully vaccinated	23 (19.66)	20 (18.02)	0.647
Boostered	81 (69.23)	80 (72.07)	0.937
Type of vaccine			0.430
Inactivated vaccine	110 (94.02)	99 (89.19)	0.233
mRNA vaccine	1 (0.85)	3 (2.70)	0.359
Adenovirus vector vaccine	0	0	1.000
Subunit protein vaccine	0	1 (0.90)	0.487
Heterologous vaccination	6 (5.13)	8 (7.21)	0.588
Date of last vaccination			0.645
Before 31 May 2022	108 (92.31)	100 (90.09)	0.642
After 1 June 2022	9 (7.69)	11 (9.91)	0.643
Body mass index (kg/m^2^)	23.8 ± 4.1	24.3 ± 4.2	0.076
HBsAg (IU/mL) [median (IQR)]	167.15 (26.23, 693.57)	321.34 (46.06, 998.03)	0.052
Cirrhosis, n (%)	10 (8.55%)	8 (7.21%)	0.808
Hypertension, n (%)	16 (13.68)	14 (12.61)	0.847
Diabetes mellitus, n (%)	12 (10.26)	10 (9.01)	0.825
Cardiovascular disease, n (%)	7 (5.98)	6 (5.41)	0.999
Dyslipidemia, n (%)	19 (16.24)	7 (6.31%)	0.022
Fatty liver, n (%)	24 (20.51)	21 (18.92)	0.868
Antiviral therapy			**< 0.001**
Entecavir	8 (6.84)	25 (22.52)	**< 0.001**
Tenofovir disoproxil fumarate	3 (2.56)	11 (9.91)	**0.013**
Tenofovir alafenamide	54 (46.15)	34 (30.63)	0.111
Tenofovir amibufenamide	23 (19.66)	41 (36.94)	**0.001**
No antiviral therapy	45 (38.46)	0	**< 0.001**

All the data were collected between December 2022 and January 2023. Values are mean ± SD, median (IQR), or number (percentage), as appropriate. SD, standard deviation; IFN, interferon;NAs, nucleoside analogs; CHB, Chronic Hepatitis B. Bold values indicate statistically significant results, defined as *p* < 0.05.

IFN treatment was associated with significantly higher levels of AST (*p* < 0.001), total bilirubin (*p <* 0.001), albumin (*p <* 0.001), eGFR (*p <* 0.001), and glucose (*p <* 0.001), as well as a significantly higher hemoglobin (*p <* 0.001) compared to *NAs* treatment. However, no significant differences were observed in GGT (*p* = 0.624), ALP (*p* = 0.357), or thrombin time (*p* = 0.228). In contrast, triglyceride levels and total cholesterol levels were significantly lower in the IFN group (*p <* 0.001). Additionally, the IFN group demonstrated significantly lower white blood cell count and platelet counts (*p <* 0.001 for all) (Table 2).

**TABLE 2:** Comparison of the baseline laboratory variables between IFN-treated and NAs-treated CHB patients infected with SARS-CoV-2 during December 2022 to January 2023 in Shanghai.

Characteristics	IFN-treated CHB patients (*n* = 117)	NAs-treated CHB patients (*n* = 111)	*P-*value
Liver function			
ALT (U/L)	27.26 (15.00, 32.00)	24.10 (14.00, 31.00)	< 0.001
AST (U/L)	25.44 (20.00, 28.00)	20.32 (16.00, 22.00)	< 0.001
GGT (U/L)	59.57 (15.00, 33.00)	55.94 (16.00, 39.05)	0.624
ALP (U/L)	136.40 (77.03, 167.00)	132.3 (99.54, 161.21)	0.357
Total bilirubin (mmoL/L)	13.05 (10.30, 15.20)	10.30 (9.77, 16.45)	< 0.001
Albumin (g/L)	42.65 (40.00, 45.00)	39.44 (37.00, 44.00)	< 0.001
Serum lipid			
Total cholesterol (mmoL/L)	4.82 (4.53, 5.13)	4.62 (3.92, 5.21)	< 0.001
Triglyceride (mmoL/L)	1.62 (1.29, 1.80)	1.73 (1.72, 1.86)	< 0.001
Renal function			
eGFR (mL/min/1.73 m^2^)	111.49 (101.78, 122,09)	98.23 (87.91, 107.25)	< 0.001
Glucose indicator (mmoL/L)	6.25 (5.56, 7.01)	5.47 (4.09, 6.98)	< 0.001
Blood routine index			
White blood cell (10^9^/L)	3.99 (2.65, 5.34)	5.63 (4.32, 7.08)	< 0.001
Hemoglobin (g/L)	128.20 (116.64, 138.98)	114.60 (104.12, 128.23)	< 0.001
Platelet (10^9^/L)	88.93 (24.03, 153.91)	177.80 (120.98, 235.76)	< 0.001
Coagulation function			
International normalized ratio	0.98 (0.93, 1.01)	0.96 (0.92, 0.98)	0.154
Thrombin time (s)	14.78 (14.30, 15.20)	14.62 (14.20, 15.12)	0.228

All the data were collected between December 2022 and January 2023. Values are median (IQR) or number (percentage). SD, standard deviation; IFN, interferon; NAs, nucleoside analogs; CHB, Chronic Hepatitis B.

### Peg-IFNα-treated CHB patients showed milder self-reported acute clinical manifestations than NAs-treated CHB patients

**FIGURE 2:**
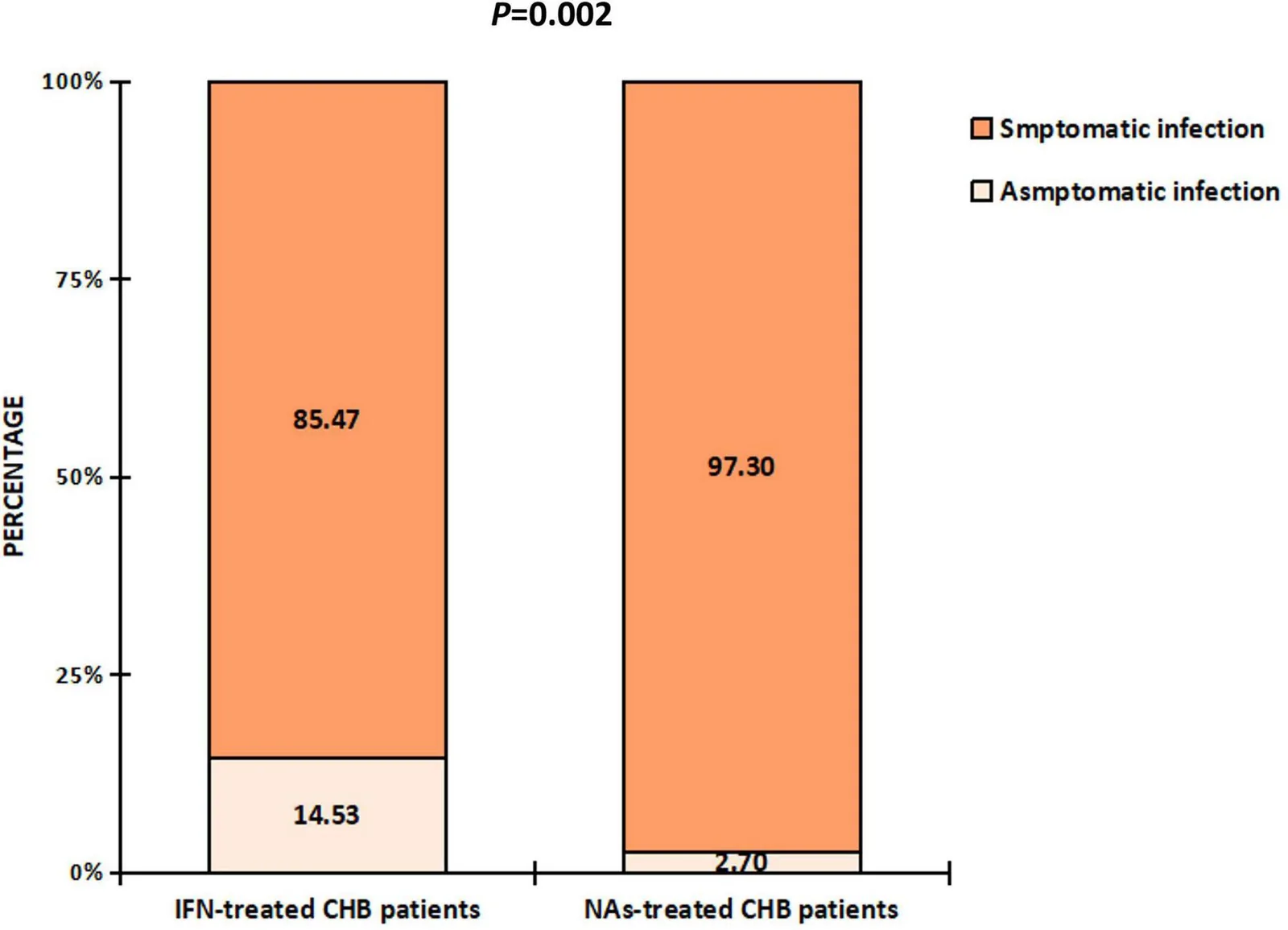
Proportion of asymptomatic SARS-CoV-2 infection among IFN-treated and NAs-treated CHB patients. The figure illustrates the proportion of asymptomatic and symptomatic SARS-CoV-2 infection among IFN-treated and NAs-treated CHB patients. The rate of asymptomatic infection was significantly higher in IFN-treated patients than in NAs-treated patients [14.53% vs. 2.70%, *P* = 0.002]. CHB, chronic hepatitis B; IFN, interferon; NAs, nucleos(t)ide analogs; SARS-CoV-2, severe acute respiratory syndrome coronavirus 2.

The proportion of asymptomatic SARS-CoV-2 infections among IFN-treated CHB patients was 14.5%, significantly higher than that observed in NAs-treated CHB patients (2.7%) (*p* = 0.002; Figure 2 and Table 3).

**TABLE 3:** Comparison of clinical characteristics between IFN-treated and NAs-treated CHB patients infected with SARS-CoV-2 during December 2022 to January 2023.

Category	IFN-treated CHB patients (*n* = 117)	NAs-treated CHB patients (*n* = 111)	*P*-value
Asymptomatic infection	17 (14.53)	3 (2.70)	**0.002**
Fever			0.296
No	27 (23.08)	25 (22.52)	
Yes, ≤ 39°C	76 (64.96)	58 (52.25)	
Yes, > 39°C	14 (11.97)	28 (25.23)	
Duration of fever (days)			**0.018**
0–1	82 (70.09)	56 (50.45)	
2–3	28 (23.93)	44 (39.64)	
4–5	3 (2.56)	7 (6.31)	
> 5	4 (3.42)	5 (4.50)	
Use of antipyretics	25 (21.36)	78 (70.27)	**< 0.0001**
Fatigue	90 (76.90)	86 (77.48)	1.000
Duration of fatigue (days)			0.793
0–3	27 (23.08)	23 (20.72)	
4–7	67 (57.26)	62 (55.86)	
8–14	12 (10.26)	10 (9.01)	
> 14	11 (9.40)	15 (13.51)	
Sore throat			1.000
No	53 (45.30)	49 (44.14)	
Mild	49 (41.88)	47 (42.34)	
Moderate	6 (5.13)	6 (5.41)	
Severe	9 (7.69)	9 (8.11)	
Cough			0.995
No	44 (37.61)	40 (36.04)	
Mild	60 (51.28)	58 (52.25)	
Moderate	10 (8.55)	10 (9.01)	
Severe	3 (2.56)	3 (2.70)	
Anosmia and/or ageusia	17 (14.53)	15 (13.51)	0.851
Muscle and/or joint pain	24 (20.51)	71 (63.96)	**< 0.0001**
Headache	30 (25.64)	70 (63.06)	**< 0.0001**
Chest congestion	5 (4.72)	12 (10.81)	0.778
Diarrhea	11 (9.40)	10 (9.01)	1.000
Runny nose	8 (6.84)	45 (40.54)	**< 0.0001**
Sleep disorders	44 (37.61)	26 (23.42)	**0.029**
CT-confirmed pneumonia	2 (1.71)	3 (2.70)	0.677

All the data were collected between December 2022 and January 2023. Data was shown as number (percentage). SD, standard deviation; IFN, interferon; NAs, nucleoside analogs; CHB, Chronic Hepatitis B. Bold values indicate statistically significant results, defined as *p* < 0.05.

**FIGURE 3:**
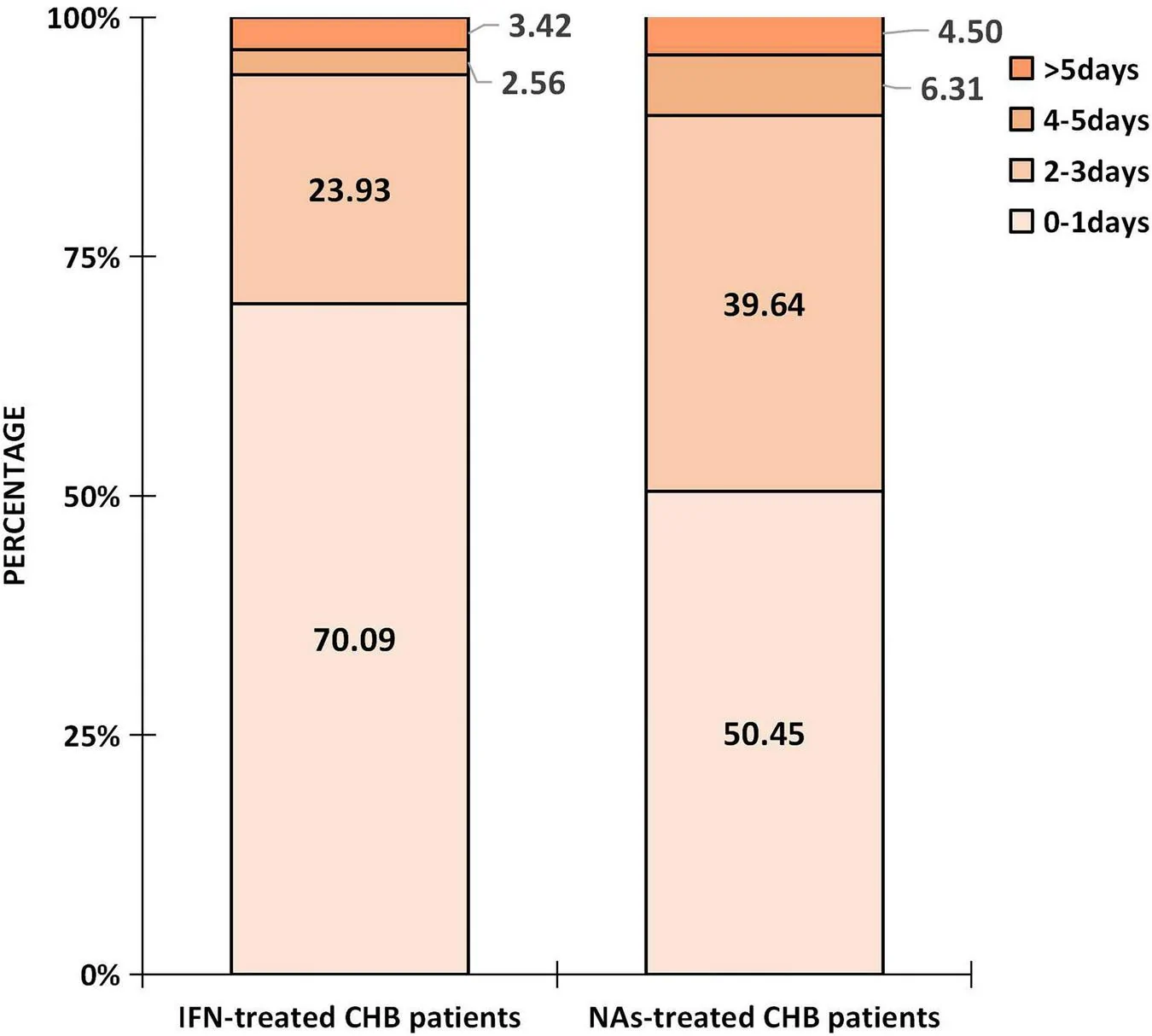
Duration of fever among IFN-treated and NAs-treated CHB patients. The figure illustrates the distribution of fever duration among IFN-treated and NAs-treated CHB patients. A higher proportion of IFN-treated patients reported fever lasting 0–1 day compared with NAs-treated patients [70.09% vs. 50.45%]. In contrast, fever lasting 2–3 days was more frequent in the NAs-treated group than in the IFN-treated group [39.64% vs. 23.93%]. The overall distribution of fever duration differed significantly between the two groups (*P* = 0.018). CHB, chronic hepatitis B; IFN, interferon; NAs, nucleos(t)ide analogs.

**FIGURE 4:**
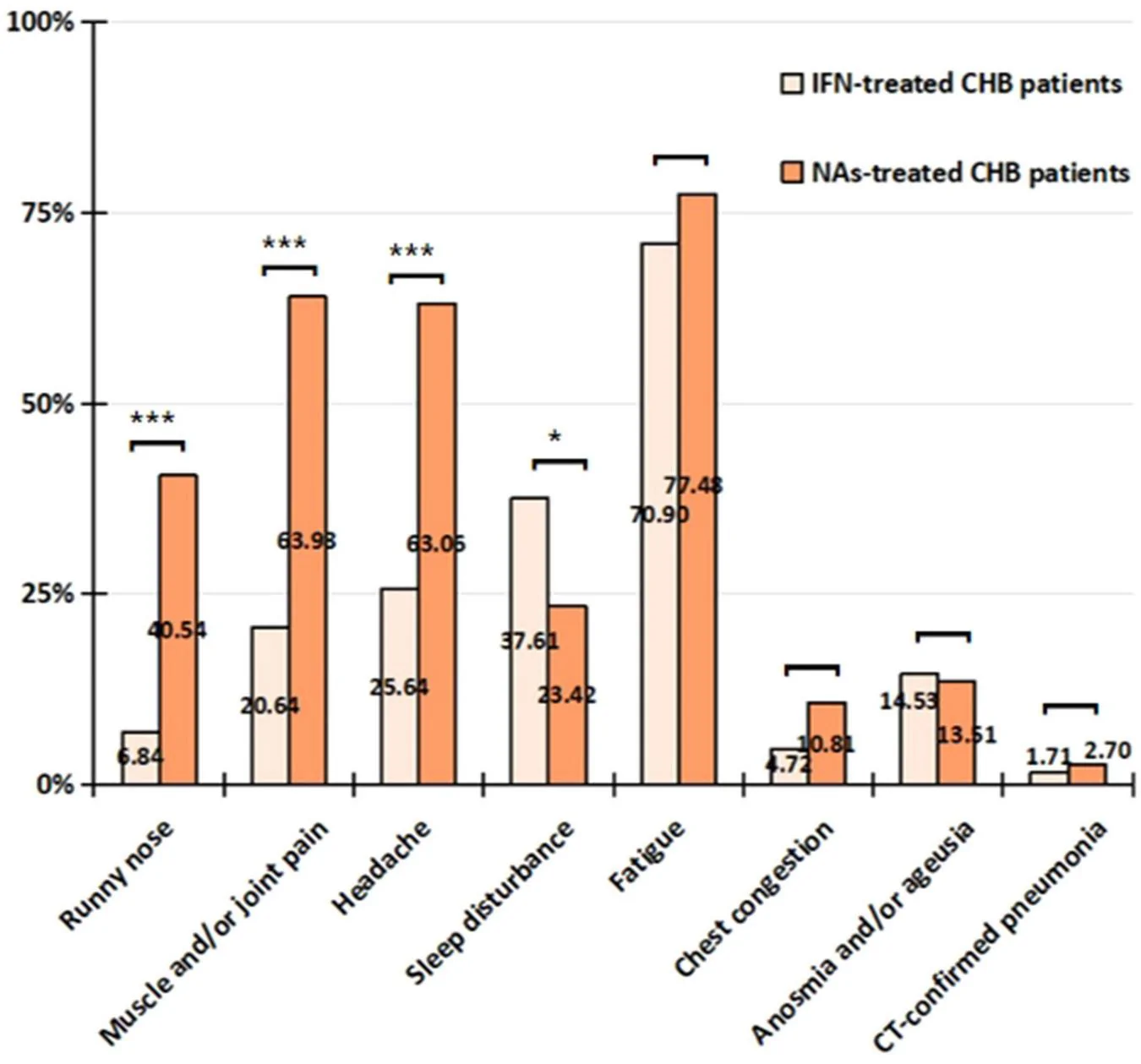
Occurrence of SARS-CoV-2 symptoms among IFN-treated and NAs-treated CHB patients. The figure presents the occurrence of SARS-CoV-2 symptoms among IFN-treated and NAs-treated CHB patients. The data indicates that IFN-treated patients showed a reduced use of antipyretics (*p* < 0.0001) and had a lower incidence of muscle and/or joint pain (*p* < 0.0001), headache (*p* < 0.0001), and runny nose (*p* < 0.0001) compared to NAs-treated patients. Notably, IFN-treated CHB patients had a significantly higher incidence of sleep disorders compared to NAs-treated patients (*p* = 0.029). The significance levels are represented as ***p < 0.001,**p < 0.01, and *p < 0.05. CHB, Chronic Hepatitis B; IFN, interferon; PEG-IFNα, Pegylated Interferon alpha; NAs, nucleoside analogs.

In addition, IFN-treated CHB patients demonstrated significantly shorter durations of fever (*p* = 0.018; Figure 3 and Table 3), reduced use of antipyretics (*p <* 0.0001), and a lower incidence of muscle and/or joint pain (*p <* 0.0001), headache (*p <* 0.0001), and runny nose (*p <* 0.0001) compared to NAs-treated CHB patients. Notably, IFN-treated CHB patients had a significantly higher incidence of sleep disorders compared to NAs-treated patients (*p* = 0.029; Figure 4 and Table 3).

The occurrence and severity of 18 commonly reported symptoms during SARS-CoV-2 infection were summarized in Table 4. The four most common symptoms of SARS-CoV-2 infection among IFN-treated CHB patients were fever (76.92%), fatigue (70.09%), cough (62.39%), and sore throat (54.70%). In contrast, the four most common symptoms of SARS-CoV-2 infection in NAs-treated CHB patients were fever (77.48%), fatigue (77.48%), muscle and/or joint pain (63.96%), and cough (63.96%) (Table 4).

**TABLE 4:** The severity of 18 Symptoms in COVID-19 infected patients.

Variable of symptom	IFN-treated CHB patients (*n* = 117)			NAs-treated CHB patients (*n* = 111)		
	N (%)	Severity	M (P25, P75)	N (%)	Severity	M (P25, P75)
Fatigue	90 (76.92)	1.06 ± 0.844	1 (1, 2)	86 (77.48)	1.08 ± 0.854	1 (1, 2)
Loss hair	4 (3.42)	0.07 ± 0.388	0 (0, 0)	4 (3.60)	0.07 ± 0.398	0 (0, 0)
Anosmia and/or ageusia	17 (14.53)	0.15 ± 0.441	0 (0, 0)	15 (13.51)	0.15 ± 0.451	0 (0, 0)
Palpitation	9 (7.69)	0.08 ± 0.268	0 (0, 0)	9 (8.11)	0.08 ± 0.274	0 (0, 0)
Muscle and/or joint pain	24 (20.51)	0.97 ± 1.038	1 (0, 1)	71 (63.96)	0.98 ± 1.036	1 (0, 1)
Decreased appetite	4 (3.42)	0.07 ± 0.338	0 (0, 0)	4(3.60)	0.07 ± 0.398	0 (0, 0)
Taste disturbance	4 (3.42)	0.07 ± 0.338	0 (0, 0)	4 (3.60)	0.07 ± 0.398	0 (0, 0)
Dizziness	52 (44.44)	0.62 ± 0.775	0 (0, 1)	50(45.05)	0.63 ± 0.785	0 (0, 1)
Diarrhea or vomiting	11 (9.40)	0.17 ± 0.562	0 (0, 0)	10 (9.01)	0.18 ± 0.575	0 (0, 0)
Sore throat or difficulty swallowing	64(54.70)	0.75 ± 0.870	0 (0, 1)	62(55.86)	0.77 ± 0.881	0 (0, 1)
Headache	30 (25.64)	0.81 ± 0.754	0 (0, 1)	70 (63.06)	0.83 ± 0.761	0 (0, 1)
Fever	90 (76.92)	1.32 ± 1.056	1 (1, 2)	86 (77.48)	1.33 ± 0.566	1 (1, 2)
Cough	73 (62.39)	0.77 ± 0.712	0 (0, 1)	71 (63.96)	0.78 ± 0.719	0 (0, 1)
Chest congestion	5 (4.27)	0.11 ± 0.342	0 (0, 0)	12 (10.81)	0.12 ± 0.350	0 (0, 0)
Runny nose	8 (6.84)	0.64 ± 0.866	0 (0, 1)	45 (40.54)	0.65 ± 0.870	0 (0, 1)
Insomnia	44 (37.61)	0.16 ± 0.508	0 (0, 0)	26 (23.42)	0.17 ± 0.520	0 (0, 0)
Lethargy	17 (14.53)	0.16 ± 0.435	0 (0, 0)	16 (14.41)	0.16 ± 0.438	0 (0, 0)
Lumbago	7 (5.98)	0.06 ± 0.238	0 (0, 0)	7 (6.31)	0.06 ± 0.244	0 (0, 0)

Values are mean ± SD or number (percentage). SD, standard deviation; IFN, interferon;NAs, nucleoside analogs; CHB, Chronic Hepatitis B. The severity is calculated according to the mean ± SD or median (IQR) of the patients’ symptom self-assessment score. Each symptom was graded as absent, mild, moderate, or severe and assigned a score of 0–3.

### Factors associated with asymptomatic SARS-CoV-2 infection among CHB patients

**FIGURE 5:**
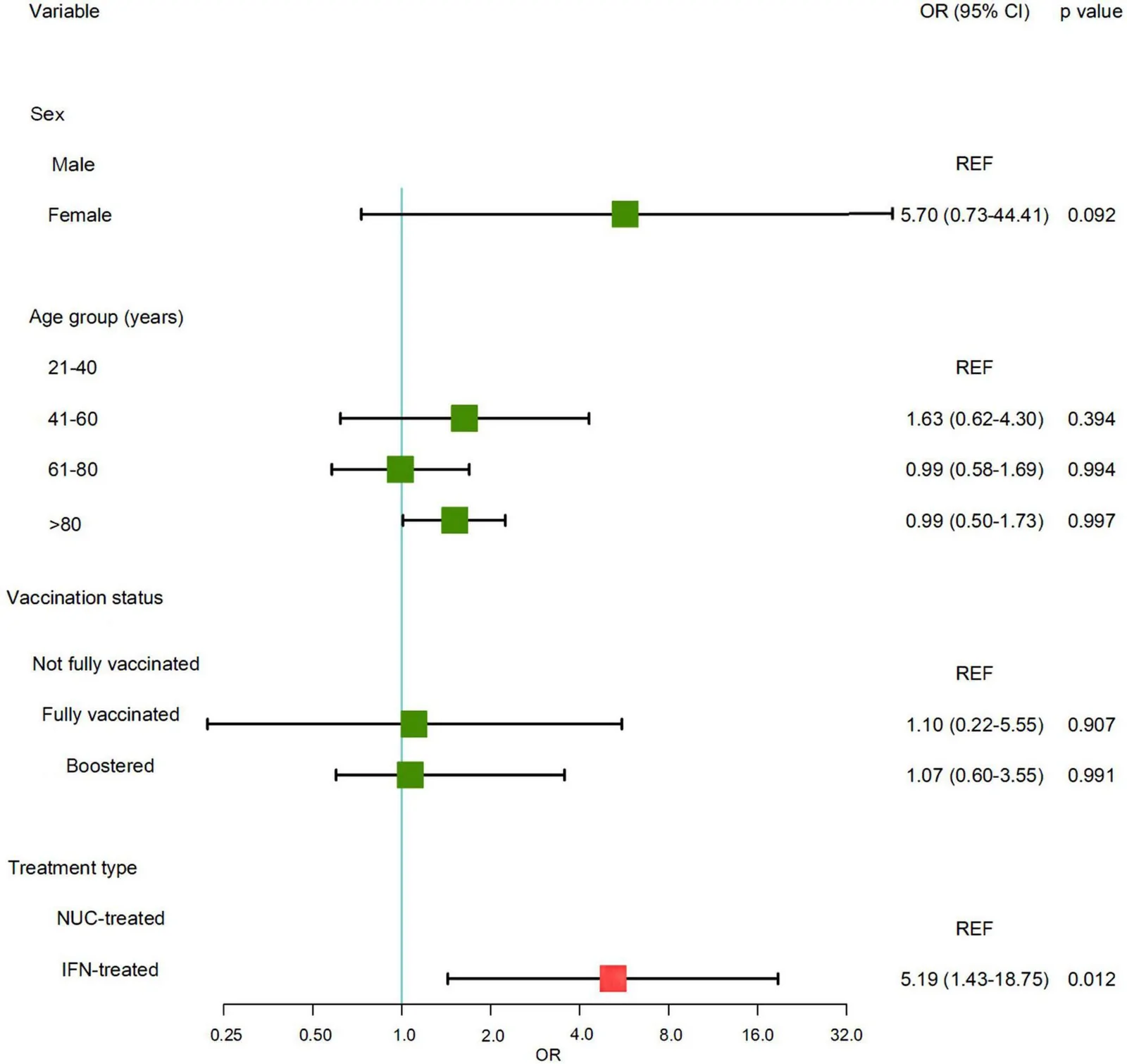
The multivariable logistic regression model for asymptomatic infections by sex, age, vaccination status and Treatment types. The figure presents the multivariable logistic regression model for asymptomatic infections by sex, age, vaccination status and Treatment types. Peg-IFNα treatment was independently associated with asymptomatic SARS-CoV-2 infection among CHB patients (aOR = 5.19, 95% CI: 1.43–18.75, *p* = 0.012). CHB, Chronic Hepatitis B; IFN, interferon; PEG-IFNα, Pegylated Interferon alpha; NAs, nucleoside analogs.

Asymptomatic infection, gender, age, vaccination status, and type of treatment were included in the multivariable analysis. IFN treatment was independently associated with asymptomatic SARS-CoV-2 infection among CHB patients (aOR = 5.19, 95% CI: 1.43–18.75, *p* = 0.012) (Figure 5 and Table 5).

**TABLE 5:** Multivariable logistic regression analysis of factors associated with asymptomatic SARS-CoV-2 infection among CHB patients.

Variable (*n* = 228)	N with asymptomatic infection	Adjust OR (95% CI)	*P-*value
Sex			
Female	1 (0.4)	Ref (Reference)	
Male	19 (8.3)	5.70 (0.73–44.41)	0.092
Age group (years)			
21–40	12 (5.3)	Ref (Reference)	
41–60	8 (3.5)	1.63 (0.62–4.30)	0.394
61–80	0	0.99 (0.58–1.69)	0.994
> 80	0	0.99 (0.50–1.73)	0.997
Vaccination status			
Not fully vaccinated	2 (0.9)	Ref (Reference)	
Fully vaccinated	18 (7.9)	1.10 (0.22–5.55)	0.907
Boostered	0	1.07 (0.60–3.55)	0.991
Treatment type			
NAs-treated	3 (1.3)	Ref (Reference)	
IFN-treated	17 (7.5)	5.19 (1.43–18.75)	**0.012**

All the data were presented as number (percentage). SD, standard deviation; IFN, interferon;NAs, nucleoside analogs; CHB, chronic hepatitis B. Bold value indicates statistically significant results, defined as *p* < 0.05.

Age group, gender, and vaccination status had no significant association with asymptomatic infection among CHB patients, yet they were included in the final model to adjust for possible confounders.

## Discussion

The proportion of SARS-CoV-2 infection among IFN-treated and NAs-treated CHB patients were high from December 2022-January 2023 in Shanghai, China (96 and 98% respectively). Peg-IFN-α treatment was independently associated with a higher proportion of asymptomatic infection among CHB patients (OR = 5.19, 95% CI: 1.43–18.75, *p* = 0.012). In this real-world observational cohort, Peg-IFNα-treated CHB patients showed milder self-reported acute clinical manifestations of SARS-CoV-2 infection than NAs-treated patients, including a higher proportion of asymptomatic infection, shorter fever duration, less use of antipyretics, and lower occurrence of several symptoms. However, because treatment allocation was not randomized, these findings should be interpreted as an association rather than evidence that Peg-IFN-α directly reduced COVID-19 severity. The only exception was that IFN-treated CHB patients had higher occurrence of sleep disorders than NAs-treated CHB patients. Our results suggest that Peg-IFN-α treatment did not prevent SARS-CoV-2 infection but was associated with milder self-reported acute clinical manifestations among CHB patients.

According to Li et al.’s study, CHB increases COVID-19 susceptibility and severity among individuals of East Asian ancestry (25, 26). Interestingly, the results of our study showed milder self-reported acute clinical manifestations of SARS-CoV-2 infection among CHB patients who received Peg-IFN-α treatments. One possible explanation is that interferon-related immune activation may influence host responses during SARS-CoV-2 infection; however, our observational design and lack of virological and inflammatory endpoints preclude mechanistic or causal conclusions. It had been reported that IFN could limit SARS-CoV-2 replication, balance ACE2 inducibility, and restrict infection of human airway epithelial cells (27). In fact, type I IFN had been under a clinical trial to treat MERS-CoV and might be further used for the treatment of COVID-19 (28). Besides, type I IFN in combination with other anti-viral drugs including lopinavir/ritonavir, ribavirin, and remdesivir had been shown to be quite effective against other coronaviruses *in vitro* (29, 30). Studies mentioned above provided theoretical bases for further usage of IFN therapy in SARS-CoV-2 infection.

The higher occurrence of sleep disorders among Peg-IFNα-treated CHB patients should be interpreted cautiously, because sleep disorders has been reported during interferon-based therapy and may therefore represent a treatment-related adverse effect rather than a COVID-19-specific manifestation (31). In the present study, the core acute symptoms of SARS-CoV-2 infection were generally similar between Peg-IFNα-treated and NAs-treated CHB patients, with fever, fatigue, cough, and sore throat being the most common symptoms. Other reported manifestations included headache, dizziness, runny nose, insomnia or difficulty falling asleep, anosmia and/or ageusia, chest congestion, lethargy, diarrhea, and lumbago. Previous studies have shown that some COVID-19-related symptoms, such as fatigue, dyspnea, chest pain, muscle pain, anosmia or ageusia, and other somatic symptoms, may persist after the acute phase of infection (32–36). However, our study did not systematically assess post-COVID condition at standardized time points after recovery. Therefore, the present findings should be interpreted as reflecting self-reported acute clinical manifestations during SARS-CoV-2 infection rather than post-COVID symptoms. Future longitudinal studies with standardized post-recovery follow-up are needed to determine whether Peg-IFNα treatment is associated with symptom persistence after COVID-19.

The study revealed that IFN treatment significantly affects clinical parameters, including liver and renal functions, and glucose metabolism, with elevated liver enzymes, total bilirubin, albumin, and eGFR, potentially reflecting both immunomodulatory effects and organ function alterations during viral infections. Similar changes in liver and renal function were also observed in a study by Chen F, which included 681 cases (37). Additionally, the results revealed that IFN treatment was associated with changes in various clinical parameters, including lower triglyceride levels and higher glucose levels, which could be attributed to its effects on lipid metabolism and stress-induced responses to viral infection (38–40). Similar effects have been observed in other studies, such as Li (40) reported alterations in metabolic and immune parameters during IFN therapy in a multicenter, retrospective cohort study of COVID-19 patients. Moreover, Hao’s preliminary matched case-control study (39) noted that IFN treatment could influence viral dynamics and host metabolism, although the exact mechanisms remain unclear. Overall, these findings underscore the complex, multifaceted effects of IFN treatment, highlighting its therapeutic potential while emphasizing the critical need for close monitoring of liver, renal, and hematological functions during therapy. These laboratory differences should not be interpreted as SARS-CoV-2-specific effects. They may reflect Peg-IFNα-related hematological or metabolic effects, baseline treatment selection, liver disease status, dyslipidemia imbalance, or acute infection-related changes. Because cirrhosis and comorbidities may influence laboratory profiles in patients with CHB, we additionally compared cirrhosis status and major comorbidities between the two groups. Cirrhosis and most measured comorbidities were comparable between Peg-IFNα-treated and NAs-treated patients, suggesting that these factors were unlikely to fully explain the observed between-group differences. However, dyslipidemia was more frequent in the Peg-IFNα group, and this baseline metabolic imbalance may partly influence lipid-related laboratory parameters. Therefore, laboratory differences, particularly serum lipid profiles, should be interpreted cautiously in the context of baseline liver disease status and comorbidities.

This is the first study to investigate the clinical characteristics of SARS-CoV-2 infection in chronic hepatitis B (CHB) patients treated with PEG-IFNα or *NAs* during December 2022-January 2023 in China. However, several limitations must be acknowledged. First, this is a single-center study, which may limit the generalizability of the findings due to a lack of diversity in the patient population. Second, the relatively small cohort size may have resulted in insufficient statistical power, increasing the potential for bias. Third, reliance on self-reported data from telephone questionnaires introduces the possibility of recall bias or inaccuracies, particularly for fever duration, medication use, and symptom severity. Fourth, symptom assessment lacked standardized evaluations before infection and after recovery. Therefore, some nonspecific symptoms may overlap with Peg-IFNα-related adverse effects, and this study could not evaluate post-COVID symptoms or long-term symptom persistence. In addition, key virological and inflammatory indicators, including HBV DNA, SARS-CoV-2 RNA clearance, CRP, and IL-6, were not systematically available, limiting our ability to assess the direct antiviral or anti-inflammatory effects of Peg-IFNα. Although 18 commonly reported symptoms were assessed, COVID-19 can involve multiple body systems, and some less common or organ-specific manifestations may not have been captured by our questionnaire. Therefore, future studies with larger sample sizes, multi-center designs, and more objective data collection methods are needed to validate and extend these findings.

## Conclusion

In conclusion, in this real-world cohort of CHB patients infected with SARS-CoV-2 during the Omicron wave, Peg-IFNα treatment was associated with milder self-reported acute clinical manifestations, including a higher proportion of asymptomatic infection and reduced occurrence of several common symptoms. These findings do not indicate that Peg-IFNα prevents SARS-CoV-2 infection, nor do they allow formal evaluation of post-COVID condition or direct antiviral efficacy against SARS-CoV-2. Further studies with prospective symptom monitoring, objective inflammatory and virological endpoints, and more comprehensive baseline characterization are needed to clarify the clinical significance of this association.

## Data Availability

The datasets presented in this study can be found in online repositories. The names of the repository/repositories and accession number(s) can be found at: https://yxky.fudan.edu.cn.
