# Correction: Peg-IFNα reduces clinical severity but cannot prevent COVID-19 infection in chronic hepatitis B patients: a cohort study in China

**DOI:** 10.3389/fmed.2026.1955510

**Published:** 2026-08-12

**Authors:** Tiantian Hu, Weien Yu, Jie Tong, Yunhui Yang, Changrong Yuan, Richeng Mao, Jiming Zhang, Jinyu Wang

**Affiliations:** 1 Shanghai Key Laboratory of Infectious Diseases and Biosafety Emergency Response, Department of Infectious Diseases, National Medical Center for Infectious Diseases, Huashan Hospital, Fudan University, Shanghai, China; 2 Key Laboratory of Medical Molecular Virology (MOE/MOH), Shanghai Institute of Infectious Diseases and Biosecurity, Shanghai Medical College, Fudan University, Shanghai, China; 3 Fudan University School of Nursing, Fudan University, Shanghai, China; 4 Department of Infectious Diseases, Jing'An Branch of Huashan Hospital, Fudan University, Shanghai, China

**Keywords:** CHB, clinical severity, HBV, PEG-IFN-α-2a, SARS-CoV-2

The funder National Key Research and Development Program of China (Grant no. 2023YFC2308500) was erroneously omitted from the **Funding** statement. The correct **Funding** statement reads:

“The author(s) declared that financial support was received for this work and/or its publication. This work was supported by the National Key Research and Development Program of China (Grant no. 2023YFC2308500), the National Natural Science Foundation of China (Grant nos. 81871640, 82172255, and 82372237), the Three-Year Initiative Plan for Strengthening Public Health System Construction in Shanghai (2023–2025) Key Discipline Project (Grant no. GWVI-11.1-09), the Shanghai Municipal Science and Technology Major Project (Grant no. ZD2021CY001), the Shanghai Shen Kang Hospital Development Center (Grant no. SHDC12019116), the Shanghai Key Clinical Specialty Construction Program (Grant no. ZK2019B24), and the Institutional Program of Huashan Hospital, Fudan University (Grant no. MX202412).”

The original version of this article has been updated.

