# Supplementary material for: Peg-IFNα reduces clinical severity but cannot prevent COVID-19 infection in chronic hepatitis B patients: a cohort study in China
Source: Front Med (Lausanne). 2026 Jul 8;13:1843714. doi: 10.3389/fmed.2026.1843714 (PMC13388745; doi:10.3389/fmed.2026.1843714)
Supplement: Supplementary file 1 [file Supplementary_file_1.docx]

**Supplementary File 1**

**COVID-19 Symptom and Vaccination Status Questionnaire**

This questionnaire was designed by the authors for this study. It includes self-assessment items related to COVID-19 symptom severity and vaccination status.

**========================================================================**

**Section 1: Personal Information**

**1. Name/Number (optional): __________________________**

**2. Age: __________ years**

**3. Gender:**

** [ ] Male**

** [ ] Female**

** [ ] Other: _____________**

**Section 2: COVID-19 Vaccination Status**

**1. Have you received any COVID-19 vaccine?**

** [ ] Not fully vaccinated**

** [ ] Fully vaccinated**

** [ ] Boostered**

*The "not fully vaccinated" group included those with no COVID - 19 vaccines or an incomplete primary vaccination per local guidelines. "Fully vaccinated" referred to individuals who completed the primary series at least 14 days before assessment, in line with local policies. The "boostered" group consisted of people who had at least one additional booster dose beyond the primary series, with the last dose taken ≥14 days prior to assessment. These vaccination status definitions followed the national public health criteria during the study.*

**2. If you have received COVID-19 vaccine(s), please specify:**

** [ ] Vaccine 1: ____________________ (Date: __________)**

** [ ] Vaccine 2: ____________________ (Date: __________)**

** [ ] Vaccine 3: ____________________ (Date: __________)**

** [ ] Booster 1: ____________________ (Date: __________)**

** [ ] Booster 2: ____________________ (Date: __________)**

**Section 3: COVID-19 Symptom Self-Assessment**

| **Question** | **Options** |
| --- | --- |
| 1. **Asymptomatic infection**   Did you develop symptoms during infection? | ☐ Yes ☐ No |
| **2. Fever** | ☐ No ☐ Yes, ≤39℃ ☐ Yes, >39℃ |
| If yes: Duration of fever (days) | ☐ 0–1 ☐ 2–3 ☐ 4–5 ☐ >5 |
| Use of antipyretics | ☐ Yes, specify: ____________ ☐ No |
| **3. Fatigue** |  |
| Fatigue duration (days) | ☐ 0–3 ☐ 4–7 ☐ 8–14 ☐ >14 |
| 1. **loss hair** | ☐ No ☐ Mild ☐ Moderate ☐ Severe |
| 1. **anosmia and/or ageusia** | ☐ No ☐ Mild ☐ Moderate ☐ Severe |
| 1. **palpitation** | ☐ No ☐ Mild ☐ Moderate ☐ Severe |
| 1. **Muscle and/or joint pain** | ☐ No ☐ Mild ☐ Moderate ☐ Severe |
| 1. **decreased appetite** | ☐ No ☐ Mild ☐ Moderate ☐ Severe |
| 1. **taste disturbance** | ☐ No ☐ Mild ☐ Moderate ☐ Severe |
| 1. **dizziness** | ☐ No ☐ Mild ☐ Moderate ☐ Severe |
| 1. **diarrhea or vomiting** | ☐ No ☐ Mild ☐ Moderate ☐ Severe |
| 1. **sore throat or difficulty swallowing** | ☐ No ☐ Mild ☐ Moderate ☐ Severe |
| 1. **headache** | ☐ No ☐ Mild ☐ Moderate ☐ Severe |
| 1. **fever** | ☐ No ☐ Mild ☐ Moderate ☐ Severe |
| 1. **cough** | ☐ No ☐ Mild ☐ Moderate ☐ Severe |
| 1. **Chest congestion** | ☐ No ☐ Mild ☐ Moderate ☐ Severe |
| 1. **runny nose** | ☐ No ☐ Mild ☐ Moderate ☐ Severe |
| 1. **insomnia** | ☐ No ☐ Mild ☐ Moderate ☐ Severe |
| 1. **lethargy** | ☐ No ☐ Mild ☐ Moderate ☐ Severe |
| 1. **lumbago** | ☐ No ☐ Mild ☐ Moderate ☐ Severe |
| **21. CT-confirmed pneumonia** | ☐ No ☐ Yes |
